# Supplementary material for: Platelet Rich Plasma and Platelet-Related Products in the Treatment of Radiculopathy—A Systematic Review of the Literature
Source: Biomedicines. 2022 Nov 4;10(11):2813. doi: 10.3390/biomedicines10112813 (PMC9687426; doi:10.3390/biomedicines10112813)
Supplement: Supplementary file 1 [file biomedicines-10-02813-s001.zip › biomedicines-1915069-supplementary.pdf]

Table S1. Product Manufacture

Author, year	PPR/PL/PRGF production
**Lemper et al. 2012[17]**	PRP with PPP
**Bhatia et al. 2016[4]**	5ml of PRP from 100ml of venous blood
**Kirchner et al. 2016[19]**	20–36 mL of venous blood + sodium citrate (3.8% weight/vol), centrifuged (PRGF system IV, Spain) at 580 g for 8 min. 2cc plasma fraction above the sedimented RBCs was collected but without aspirating the buffy coat platelets. PRGF was activated by 20 µL of PRGF activator (10% calcium chloride) per ml of PRGF.
**Centeno et al. 2017[6]**	PL: 60 cc of blood centrifugated at 200 g for 10 min. 3-10cc of supernatant placed in − 80° C for 5–10 min and thawed. Then re-centrifuged to separate remaining platelets, supernatant was injected or frozen.
**Correa et al. 2019[20]**	PRGF
**Rawson et al. 2019[18]**	PRP: 180 mL of whole blood by double centrifugation) processed to produce PL by mechanical rupture followed by filtration to remove cellular debris. PRP diluted in PPP 4 to 6 times to treat the posterior structures (spinal ligaments, facet joints).
**Bise et al. 2020[21]**	2.5ml of PRP: 27ml of venous blood +3 mL of anticoagulant centrifuged at 620 g for 15 min, PLT 520,000/mm3 ± 114,250, WBC 310/mm3 ± 293
**Xu et al. 2021[22]**	PRP: 3cc of PRP: 18ml of venous blood +2 ml of 3.8% (w/v) sodium citrate. Centrifuged at 1600 rpm for 10 mins, RBCs were removed, centrifuged again at 3200 rpm for 10 minutes at RT. 4 ml was collected from the lower part which contains PRP.
**Ruiz-Lopez et al, 2021[23]**	LR-PRP: LR-PRP 16.5ml: 60 ml of blood centrifuged 14 mins 1568g, +5 mL of acid citrate dextrose, upper and intermediate level collected as LR-PRP
**Benítez Núñez et al., 2021[24]**	5mL of autologous ozonized PRP or 5mL mixture including bupivacaine and 40mg of triamcinolone
**Kirchner et al. 2021[25]**	72 mL of blood in 9-mL tubes with sodium citrate (3.8% wt/vol) (Endoret Traumatology kit, BTI), centrifuged in the PRGF Endoret System centrifuge. 2 mL of plasma fraction above the buffy coat was collected. Activation of PRGF by adding PRGF activator (10% calcium chloride).
**Machado et al. 2021[26]**	2 ml of PRP for foraminal injections, 5 ml for caudal epidural injection, (2 ml for each facet joint and capsule, 2 ml for each site of paravertebral muscles and 1 ml for intradiscal injection)
